# Prostaglandin D2/J2 signaling pathway in a rat model of neuroinflammation displaying progressive parkinsonian-like pathology: potential novel therapeutic targets

**DOI:** 10.1186/s12974-018-1305-3

**Published:** 2018-09-20

**Authors:** Chuhyon Corwin, Anastasia Nikolopoulou, Allen L. Pan, Mariela Nunez-Santos, Shankar Vallabhajosula, Peter Serrano, John Babich, Maria E. Figueiredo-Pereira

**Affiliations:** 1 Department of Biological Sciences, Hunter College, Neuroscience Collaborative Program, Graduate Center, The City University of New York, 695 Park Ave., New York, NY 10065 USA; 2 Department of Psychology, Hunter College, The City University of New York, New York, NY USA; 3 Department of Radiology, Weill Cornell Medicine, New York, NY USA

**Keywords:** Neuroinflammation, Cyclooxygenase, Prostaglandins, L-PGDS, DP2, 15-PGDH, Parkinson’s disease, PGD2/J2 signaling, Ibuprofen

## Abstract

**Background:**

Prostaglandins are products of the cyclooxygenase pathway, which is implicated in Parkinson’s disease (PD). Limited knowledge is available on mechanisms by which prostaglandins contribute to PD neurodegeneration. To address this gap, we focused on the prostaglandin PGD2/J2 signaling pathway, because PGD2 is the most abundant prostaglandin in the brain, and the one that increases the most under pathological conditions. Moreover, PGJ2 is spontaneously derived from PGD2.

**Methods:**

In this study, we determined in rats the impact of unilateral nigral PGJ2-microinfusions on COX-2, lipocalin-type PGD2 synthase (L-PGDS), PGD2/J2 receptor 2 (DP2), and 15 hydroxyprostaglandin dehydrogenase (15-PGDH). Nigral dopaminergic (DA) and microglial distribution and expression levels of these key factors of the prostaglandin D2/J2 pathway were evaluated by immunohistochemistry. PGJ2-induced motor deficits were assessed with the cylinder test. We also determined whether oral treatment with ibuprofen improved the PD-like pathology induced by PGJ2.

**Results:**

PGJ2 treatment induced progressive PD-like pathology in the rats. Concomitant with DA neuronal loss in the substantia nigra pars compacta (SNpc), PGJ2-treated rats exhibited microglia and astrocyte activation and motor deficits. In DA neurons, COX-2, L-PGDS, and 15-PGDH levels increased significantly in PGJ2-treated rats compared to controls, while DP2 receptor levels were unchanged. In microglia, DP2 receptors were basically non-detectable, while COX-2 and L-PGDS levels increased upon PGJ2-treatment, and 15-PGDH remained unchanged. 15-PGDH was also detected in oligodendrocytes. Notably, ibuprofen prevented most PGJ2-induced PD-like pathology.

**Conclusions:**

The PGJ2-induced rat model develops progressive PD pathology, which is a hard-to-mimic aspect of this disorder. Moreover, prevention of most PGJ2-induced PD-like pathology with ibuprofen suggests a positive feedback mechanism between PGJ2 and COX-2 that could lead to chronic neuroinflammation. Notably, this is the first study that analyzes the nigral dopaminergic and microglial distribution and levels of factors of the PGD2/J2 signaling pathway in rodents. Our findings support the notions that upregulation of COX-2 and L-PGDS may be important in the PGJ2 evoked PD-like pathology, and that neuronal DP2 receptor antagonists and L-PGDS inhibitors may be novel pharmacotherapeutics to relieve neuroinflammation-mediated neurodegeneration in PD, circumventing the adverse side effects of cyclooxygenase inhibitors.

## Background

Neuroinflammation is activated upon CNS injury to acutely initiate repair mechanisms, while chronic neuroinflammation can exacerbate, spread, and prolong CNS injury. Chronic neuroinflammation plays a central role in PD [1–5]. Critical to neuroinflammation is cyclooxygenase-2 (COX-2), which is highly induced in PD and negatively affects neurons [6–8].

As far as we know, there are no models of PD that utilize endogenous products of cyclooxygenases to mimic the pathology of PD [9], besides our previous studies with mice [10, 11]. PGD2 is the most abundant prostaglandin in the brain [12–15], and it raises the most under pathological conditions [16]. PGD2 is highly unstable (estimated brain half-life of 1.1 min) [17], leading to spontaneous non-enzymatic formation of PGJ2 [18]. Our rationale for using PGJ2 to establish a rat model of neuroinflammation is that we found that PGJ2 is highly neurotoxic compared to PGD2 and PGE2 [19]. In contrast to PGD2 and PGE2, PGJ2 and its metabolite 15d-PGJ2 bind covalently to proteins through their α,β-unsaturated carbonyl groups [18]. In addition, J2 prostaglandins are uptaken by cells via a carrier-mediated active transport, ending up in the cytoplasm and nucleus [20]. This endocytic transport is unique to J2 prostaglandins, as it does not apply to PGD2 and E2 [20]. PGJ2 derivatives seem to activate the nuclear peroxisomal proliferator activator receptor (PPARγ) [14], and PPARγ agonists are being cautiously tested as anti-inflammatory drugs to treated PD [21].

In rodents, the brain levels of PGJ2 are highly induced upon stroke (cerebral ischemia) [15] and traumatic brain injury (TBI) [22] reaching concentrations that are neurotoxic. Moreover, both stroke and TBI increase the long-term risk for PD [23, 24]. We recently showed that PGJ2-microinfusions into the brain induce PD-like pathology in mice [10, 11].

In neurons, PGJ2 upregulates COX-2 [25] potentially establishing a positive feedback loop between PGJ2 and COX-2 that could promote the transition from acute to chronic neuroinflammation. To investigate whether inhibiting cyclooxygenases diminishes the progressive PD-like pathology induced by PGJ2, we administered ibuprofen orally with food, to control (DMSO) and PGJ2-treated rats. Ibuprofen is a non-steroidal anti-inflammatory drug (NSAID) that inhibits COX-1 and COX-2, thus reducing the synthesis of all prostaglandins [1]. Epidemiological studies support an inverse relation between the use of ibuprofen and the risk of developing PD [26, 27], with the risk reduction being by as much as 50% [28]. However, clinical studies with NSAIDs have not shown a similar trend, specifically for Alzheimer’s disease (AD) [2, 28]. Thus, a better understanding of the shared and diverse mechanism of action of NSAIDs is required.

Rat models offer many advantages over mouse models for human disease [29]. Rats share 90% of their genome with humans, and most currently identified disease-linked human genes have equivalent ones within the rat genome [30]. Compared to the mouse, rat physiology is easier to monitor and closer to the human condition, and rats are capable of learning a broader variety of tasks [29]. Rats are also larger than mice and thus better suited for surgeries and for use in the small-animal PET system (μPET) in preclinical research and drug development, which requires high spatial resolution.

In this study, we focused on the PGJ2-induced rat model of neuroinflammation that exhibits progressive PD-like pathology including dopaminergic neuronal loss in the SNpc, microglia and astrocyte activation, and motor deficits. Oral treatment with ibuprofen prevented most PGJ2-induced PD-like pathology, indicating a potential positive feedback loop between PGJ2 and COX-2. Our analysis also suggests that upregulation of COX-2 and L-PGDS could be important in the PGJ2 evoked PD-like pathology. This pre-clinical rat model that we developed could be highly valuable to identify biomarkers and optimize therapeutics, such as DP2 antagonists or L-PGDS inhibitors, to diminish neuroinflammation in PD, circumventing the adverse side effects of cyclooxygenase inhibitors.

## Methods

### Reagents and antibodies

PGJ2 (cat. # 18500) and ibuprofen (cat. # 70280) from Cayman Chemical (Ann Arbor, MI). PGJ2 (33.4 μg/injection) was diluted in DMSO and then further diluted in PBS to a final DMSO concentration of 17% for PGJ2 microinfusions. The PGJ2 solutions were freshly prepared and stored for a maximum of 2 h at 4 °C and in the dark. A 17% DMSO/PBS was microinfused as the vehicle control. Primary antibodies were: dopaminergic neurons [TH, tyrosine hydroxylase, 1:1000, cat.# MAB318 (mouse, Millipore, Billerica, MA) and cat.# Ab6211 (rabbit, Abcam, Cambridge, MA)]; microglia [Iba1, 1:500, cat.# 019-19741 (rabbit, Wako, Richmond, VA) and 1:1000, cat.# ab139590 (chicken, Abcam)]; astrocytes [GFAP, 1:1000, cat.# AB5541 (chicken, Millipore)]; oligodendrocytes [GST-pi, 1:200, cat.# ab53943 (goat, Abcam)]; polyubiquitinated proteins [Ub, 1:200, cat.# BML-PW8805 (mouse, Enzo Life Sciences, Farmingdale, NY)]; phosphoS129 α-synuclein [pS129 α-syn, 1:200, cat.# p1571-129 (rabbit, PhosphoSolutions, Aurora, CO)]; cyclooxygenase-2 [COX-2, 1:250, cat.# 160106 (mouse, Cayman Chemical)]; prostaglandin D synthase [L-PGDS, 1:200, cat.# ab182141 (rabbit, Abcam)]; prostaglandin D2 receptor [DP2, 1:1000, cat.# PA5-20332 (rabbit, ThermoFisher, Waltham, MA)]; 15-Hydroxyprostaglandin dehydrogenase [15-PGDH, 1:500, cat.# NB200-179, (rabbit, Novus, Littleton, CO)]. Secondary antibodies—Alexa Fluor 568 (1:250, cat.# A11036, rabbit and cat.# A11031, mouse), Alexa Fluor 488 (1:250, cat.# A11039, chicken), and Alexa Fluor 350 (1:250, cat.# A10039, rabbit and cat.# A10035, mouse)—were from Life Technologies (Carlsbad, CA). Vectashield mounting medium (cat# H-1000, Vector Laboratories, Burlingame, CA).

### Rats

Sixteen-week-old Sprague Dawley male rats (*N* = 49; body weight 376–533 g) were obtained from Taconic Biosciences (Hudson, NY) and Envigo (Frederick, MD). Rats were singly housed on a 12-h light/dark cycle, maintained at 23 °C and 50–70% humidity, with food and water available ad libitum. Rats were allowed to acclimate for 1 week before the baseline behavior assessment and were 18 weeks old at the time of the first injection.

### Surgery and microinfusions

We followed the same procedures for the rats as described in our previous study with mice [11]. Rats received unilateral (right side) injections of vehicle (DMSO) or PGJ2 into the SN. Briefly, at 18 weeks of age, rats were anesthetized by isoflurane inhalation (induction 2.5–3.5%, maintenance 2.5%) administered in 100% oxygen and placed into a digital stereotaxic instrument (Model 51730D, Stoelting Co., Wood Dale, IL) fitted with a rat anesthesia mask (Model 906, David Kopf Instruments). A burr hole was drilled in the skull at coordinates for the SNpc [31]: anterior-posterior (AP) = − 5.6 mm; medial-lateral (ML) = + 2.0 mm; and dorsal-ventral (DV) = − 8.0 mm relative to the bregma. All injections were administered to the right SN, while the contralateral (left) side served as an internal control. A 2-μl microinjection Hamilton syringe (7002 KH) with a 25-gauge needle was slowly inserted into the brain and left in place for 2 min. Thereafter, 2 μl of solution was infused at an injection rate of 0.2 μl/min (Quintessential stereotaxic injector, Model 53311, Stoelting Co.). The needle was left in place an additional 3 min to ensure total diffusion of the solution. Following injection, the needle was slowly removed and the incision was closed with monofilament absorbable sutures (cat. # 038729; Henry Schein, Melville, NY). After surgery, rats were removed from the stereotaxic frame and administered a subcutaneous injection of 1 cc Lactated Ringer’s solution, given wet palatable rodent chow, and kept in a warm place to recover. Subsequent injections to the SN were administered via the same drill hole established during the first surgical procedure.

### Ibuprofen administration

Ibuprofen can be administered to rats twice daily for up to 80 days without adverse secondary side effects [32]. In our experiments rats were fed ibuprofen fortified chow (ibuprofen: ~ 40 mg / kg body weight) chronically starting the day after the first PGJ2 treatment. Ibuprofen was added to Purina 5001 Rodent Chow (800 ppm, Research Diets, Inc., New Brunswick, NJ). Individual intake of ibuprofen was measured daily by weighing the given and leftover chow per animal.

### Experimental design

**Fig. 1:**
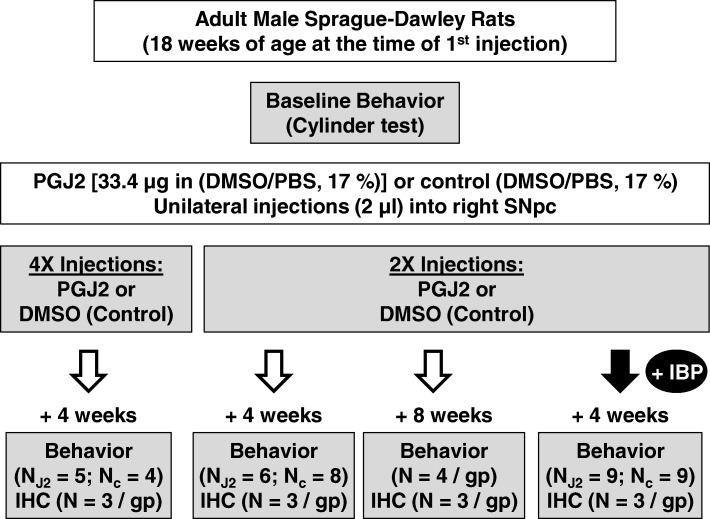
Schematic representation of the experimental design for the rat PGJ2-treatment. Eighteen-week-old male Sprague Dawley male rats were microinjected into the right SNpc as shown. Rats received two or four microinfusions of either DMSO (control; 17% in PBS) or PGJ2 (33.4 μg) in 2 μL DMSO/PBS at weekly intervals. Ibuprofen (IBP)-treated rats were fed IBP-mixed chow (800 ppm, ~ 40 mg/kg body weight) starting the day after the first microinfusion and ending 5 weeks later. For all rats, motor behavior was assessed before the first injection and 4 weeks or 8 weeks after the last injection. Following post-injection behavioral assessment, rats were perfused intracardially and the brains were removed for immunohistochemical analyses. *N* = number of rats per group (gp). X = number of injections (once per week)

Rats in each group received two or four unilateral DMSO or PGJ2 injections (once / week for two or four consecutive weeks), starting at the age of 18 weeks. Rats were randomly assigned to eight groups receiving the following microinfusions (Fig. 1): (1) four × DMSO alone (*n* = 4), sacrificed 4 weeks after the last injection; (2) four × DMSO/PGJ2 (*n* = 5), sacrificed 4 weeks after the last injection; (3) two × DMSO alone (*n* = 8), sacrificed 4 weeks after the last injection; (4) two × DMSO/PGJ2 (*n* = 6), sacrificed 4 weeks after the last injection; (5) two × DMSO alone (*n* = 4), sacrificed 8 weeks after the last injection; (6) two × DMSO/PGJ2 (*n* = 4), sacrificed 8 weeks after the last injection; (7) two x DMSO fed ibuprofen (*n* = 9), sacrificed 4 weeks after the last injection; (8) two × DMSO/PGJ2 fed ibuprofen (*n* = 9), sacrificed 4 weeks after the last injection.

#### Rationale

Our first experimental group that received four PGJ2 injections and was sacrificed 4 weeks after the last injection, exhibited severe dopaminergic (DA) neuronal loss in the injected side of the SNpc, compared to the control DMSO-treated rats. In these rats, the motor deficits in the PGJ2 group were highly significant compared to its control group. Since the dopamine levels in the brains of PD patients are reduced by 70–80% when their motor symptoms occur [33], our PGJ2 rat model with four injections successfully replicated the two major aspects of PD: DA neuronal loss with concomitant motor deficits. We also aimed at modeling the mild/early pathology that worsens progressively, because the early stage of PD is challenging to detect as it develops progressively without any validated biomarkers. Thus, we used a two-injection paradigm. In addition, as epidemiological studies report a beneficial effect of NSAIDs in lowering the risk of developing PD [26], we were interested in testing whether ibuprofen-treatment would prevent/minimize the mild/early pathology detected in our two-injection PGJ2 rat model.

### Behavior

Rats were tested for parkinsonian-like behavior 4 or 8 weeks after their last injection (Fig. 1). We used the cylinder behavioral test that assesses spontaneous activity and limb use asymmetry in a novel environment. This is considered a highly reliable and sensitive behavioral test used to detect unilateral damage to the nigrostriatal pathway [34]. An additional advantage of the cylinder test is that inter-rater reliability is very high (*r* > 0.95) even with relatively inexperienced raters [34, 35].

Rats were placed in a transparent cylinder (11 in. in diameter and 18 in. in height) and video-recorded for 5–10 min depending on the activity level. A mirror was placed at an angle underneath the raised cylinder to allow the view of the back wall and the bottom of the cylinder. To determine the extent of asymmetry, the use of the left and right forelimb was counted during the wall movements after a rear, and during the landings as described in [34].

Forelimb use asymmetry is determined by the preference toward the non-impaired forelimb for weight shifting during spontaneous vertical exploration and landing. These movements along the wall and landings after a rear, were separately recorded and then averaged for total asymmetry, to correct for variability as described in [34, 35]. Each cylinder video was scored by two raters blind to the experimental group to further reduce the variability. Their scores were averaged and statistically analyzed.

Rodents display innate limb preference correlated to endogenous nigrostriatal DA imbalance [36]. Thus, the effect of the unilateral surgery on the behavior was measured as percent (%) change of the forelimb use asymmetry post-surgery, compared to the baseline behavior pre-surgery.

### Immunohistochemistry

After the behavior analyses, rats were anesthetized (i.p.) with ketamine (100 mg/kg) and xylazine (5 mg/kg), and transcardially perfused with 4% paraformaldehyde in PBS. The rat brains were removed, post-fixed overnight at 4 °C, followed by cryoprotection (30% sucrose/PBS at 4 °C). Brains were sectioned in the coronal plane using a freezing microtome at a thickness of 30 μm, and sections were collected serially along the anterioposterior axis of the SNpc [between − 4.56 mm and − 6.36 mm for the SNpc; coordinates relative to bregma, as in [31]]. Tissue series (eight series for the SNpc) were stored at 4 °C in cryoprotectant (30% glycerol and ethylene glycol in PBS) until use. Sections were processed free floating for immunohistochemical (IHC) analyses as described in [10]. At the end, sections were mounted on gelatin-subbed glass slides with Vectashield. Sections were viewed under a wide-field fluorescence microscope (Zeiss AxioImager) using a Zeiss AxioCam MRm Rev. 3 camera connected to a motorized stage; the software AxioVision 4 module MosaiX was used to capture whole SN region mosaics (× 10 magnification).

Exposure time for each channel was kept consistent between sections. For each captured image, ZVI files were loaded onto Image J (NIH, Bethesda, MD) and converted to .tif files for use in optical density and co-localization analyses. Each channel was analyzed to an antibody specific threshold [37]. Pixel areas meeting threshold intensity criteria were measured in delineated SN regions.

The SNpc was delineated based on tyrosine hydroxylase (TH+) staining at × 10 magnification while carefully excluding cells within the ventral tegmental area (VTA).

For optical density (O.D.) analysis, three tissue sections per treatment group were used for quantification. Nonspecific background density was corrected using ImageJ rolling-ball method [38]. Data was expressed as O. D. ratio from the ipsilateral SNpc over the contralateral.

### Microglia analysis

Activated microglia exhibit a remarkable variety of morphologies that can be associated with their particular functions [39]. Using binary images of individual microglia silhouettes, we distributed activated microglia into three different groups according to their form factor (FF) value (Fig. 4c), which is defined as 4π X area/perimeter^2^ [40]. Each of the three microglia groups is defined as follows [39]: *Ramified*, FF: 0 to 0.5; actively engaged in neuronal maintenance providing neurotrophic factors. *Reactive*, FF: > 0.5 to 0.7; responsive to CNS injury. *Amoeboid*, FF > 0.7 to 1; cell body is amorphous with pseudopodia.

### Stereology

Unbiased stereology analysis was conducted by scorers blind to the experimental treatments as described in [11]. Total number of TH+ and Iba1+ cells were obtained with the Zeiss AxioVision Release 4.8.2 Software unbiased stereology programming (Carl Zeiss Group, Jena, Germany). The outline of the SNpc delineated by TH+ staining [41] was obtained at low (× 10) magnification. Within the delineated SNpc area, depending on the anterioposterior position of the coronal section, 5–11 sites were sampled with the optical fractionator probe. For each brain, six SNpc sections spaced 240 μm apart were used for the analyses. Cells were counted at × 40 magnification along the AP axis, and at predetermined intervals [x-step = 230 μm; y-step = 170 μm; 39,100 μm^2^; frame associated area (grid size): horizontal = 226.057 μm, vertical = 168.380 μm; 38,063.478 μm^2^]. The *z*-depth was set at 20 μm to allow a 5-μm guard on the top and bottom surface of each section to avoid error in case of tissue damage. Estimated cell numbers in the whole SNpc were used for group comparison. Dopaminergic (DA) cell counts from the intact SNpc were similar to a previous report [42]. Care was taken to ensure that cells within the VTA were excluded from SNpc quantification.

### Statistical analyses

All data are expressed as the mean ± SEM. Statistical analyses were performed with GraphPad Prism 6 (GraphPad Software, San Diego, CA). A *p* value < 0.05 was considered statistically significant. For group comparisons, we performed one-way analysis of variance (ANOVA) followed by post hoc Tukey’s test for multiple comparison. One-tailed Student’s *T* test was used to compare means between two groups*.* Correlations between two variables were evaluated by linear regression calculating the Pearson correlation coefficients.

## Results

### PGJ2 induces progressive dopaminergic neuronal loss in the rat SNpc

Following our recently established PGJ2-induced mouse model of PD-like pathology [10, 11], we investigated in vivo the progressive effects of subchronic inflammation in rats. For this purpose, rats were administered unilateral (right side) injections of PGJ2 to the SNpc for 2 or 4 weeks (once per week) as depicted in Fig. 1. Following behavioral analysis, the rats were sacrificed 4 or 8 weeks post the last injection, and their brains analyzed by immunohistochemistry.

To assess DA-specific neuronal damage, we performed IHC staining for TH+. We used the unbiased optical fractionator stereological method and compared the ratio between the numbers of TH+ cells in the ipsilateral SNpc over that in the contralateral side for each rat.

**Fig. 2:**
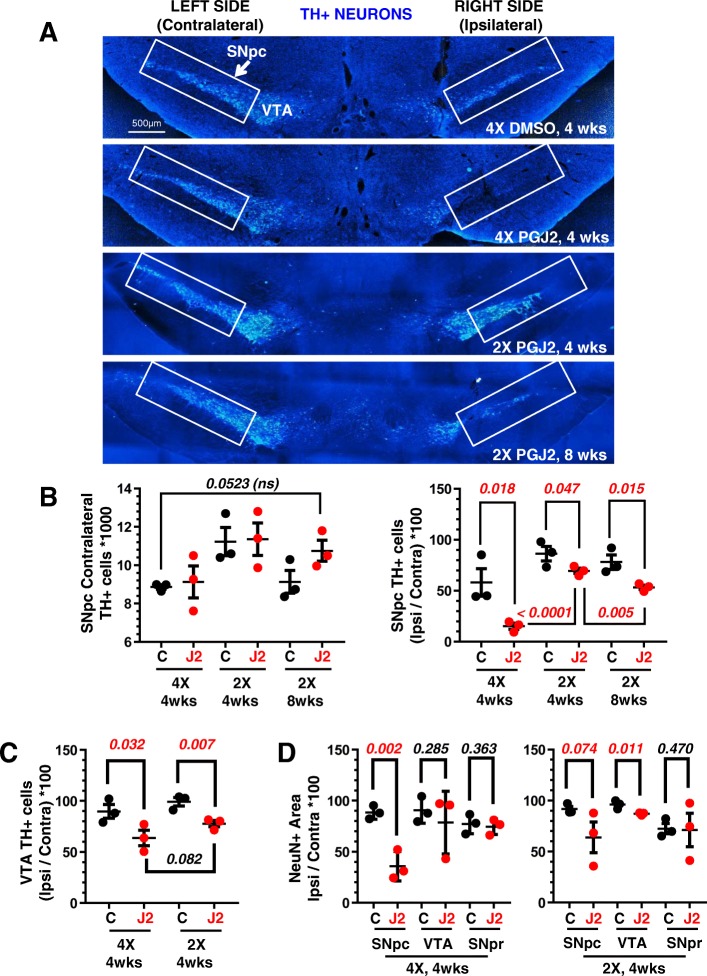
Progressive dopaminergic neuronal loss in the SNpc upon successive intranigral PGJ2 microinjections. **a** Representative coronal sections of the ventral midbrain depicting dopaminergic neurons. TH-immunoreactivity was strong in the VTA and SNpc (rectangle) of control (DMSO) rats. TH-immunoreactivity decreased in the ipsilateral SNpc of rats receiving two (2X) or four (4X) PGJ2 injections. Dopaminergic neurons in the VTA were less affected. Scale bar = 500 μm. **b** The extent of PGJ2-induced damage was assessed by calculating the total number of TH+ neurons (mean ± SEM) in the SNpc using unbiased stereology as described in Materials and Methods. No significant differences in TH+ neurons were observed in the contralateral SNpc of the different groups of rats. TH+-immunoreactivity decreased in a gradual manner in the ipsilateral SNpc of rats receiving two (2X) or four (4X) PGJ2 injections and at 4 weeks or 8 weeks post-injections of two (2X) PGJ2 injections. **c** PGJ2-induced damage in the VTA was measured by TH+ area (mean ± SEM). Dopaminergic neurons in the VTA were less affected than in the SNpc. **d** Neuronal loss in the SNpc, VTA, and SNpr were compared by NeuN+ area (mean ± SEM). NeuN-immunoreactivity decreased significantly in the SNpc, but less in the VTA and not significantly in the SNpr of PGJ2-injected rats compared to controls (DMSO-treated). Black circles, control, DMSO-treated rats; red circles, PGJ2-treated rats. Statistical significance was estimated with one-way ANOVA (**b**, left), and the Student’s *T* test (**b**, right, **c** and **d**) to compare DMSO and PGJ2-treated groups and between two PGJ2-treated groups. The *p* values in red indicate statistical significant (< 0.05) difference from DMSO-injected rats. *N* = 3 rats per group. X = number of injections (once per week)

Rats, treated with four PGJ2-microinfusions and sacrificed 4 weeks following the last injection, displayed intense (74%) DA neuronal loss in the ipsilateral SNpc (Fig. 2a, second panel, and 2b, right panel), compared to DMSO-treated rats. We thus decided to reduce the number of injections to two, with the hope of detecting progressive DA neuronal loss. Indeed, DA neuronal loss was significantly less upon two than four PGJ2 injections (Fig. 2a, third and fourth panels, and 2b, right panel). Moreover, DA neuronal loss was progressive, as it significantly [*t*(*4*) = 4.65; *p =* 0.005] intensified 8 weeks compared to 4 weeks post-injection. Compared to DMSO-treatment, DA neuronal loss was 32% and 20% respectively, 8 and 4 weeks following PGJ2-treatment. The contralateral SNpc served as the uninjected control for each rat, and no significant differences were detected among groups (Fig. 2a, b, left panel).

The DA neurons in the VTA were less affected than those in the SNpc (Fig. 2c). Immunostaining for neuron-specific nuclear protein (NeuN) decreased significantly in the SNpc, following PGJ2 administration [*t*(4) = 5.76; *p* = 0.002], thus corroborating that the loss in TH immunostaining was due to the degeneration of DA neurons and not to TH depletion (Fig. 2d, left panel). The decrease in NeuN+ immunoreactivity in the VTA and SN pars reticulata (SNpr) was either non-significant or less than in the SNpc (Fig. 2d, right panel).

### PGJ2-treated rats develop parkinsonian-like motor deficits in a progressive manner

Clinically, PD is characterized by the development of motor deficits caused by the loss of dopamine in the SNpc [43–45]. To determine the effect of PGJ2 treatment on motor performance, we measured the forelimb use asymmetry with the cylinder test which is considered a sensitive measure of the degree of unilateral nigrostriatal damage [46]. Unilateral injections into the right SN should cause forelimb impairment in the contralateral (left) side and encourage the use of the ipsilateral, right-forelimb, thus reflecting a change in nigrostriatal DA. For each animal, the percent (%) change in asymmetry between pre (baseline) and post-injection was calculated. The mean ± SEM for each treatment group was used for statistical analysis.

**Fig. 3:**
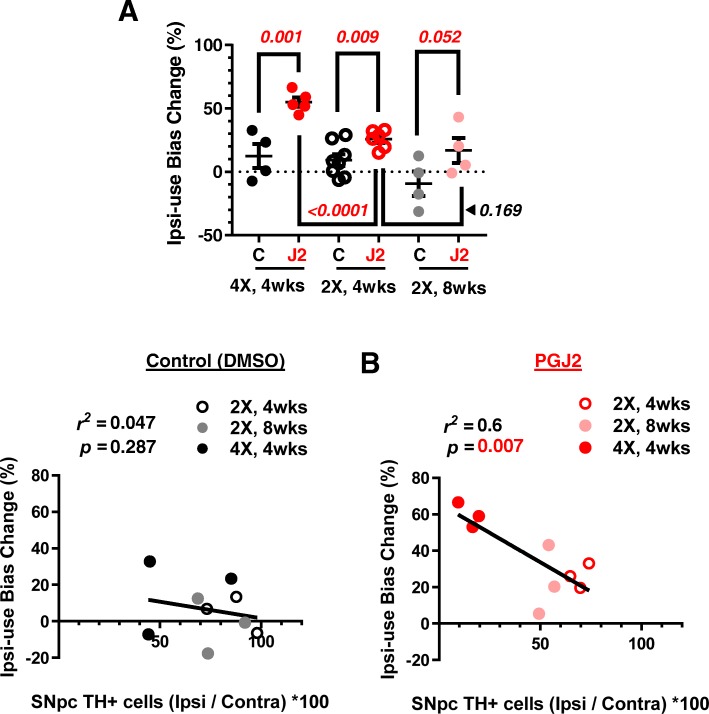
Correlation between behavioral deficits and dopaminergic (TH+) neuronal loss in the SNpc induced by successive PGJ2 microinfusions. **a** All groups of rats treated with PGJ2 (shades of red circles) exhibited behavioral deficits. When compared to DMSO-treated rats (white, gray and black circles), those treated with 2X and 4X PGJ2 significantly increased usage of the ipsilateral forelimb as assessed by the cylinder test (see Materials and Methods). Statistical significance was estimated with the Student’s *T* test to compare DMSO and PGJ2-treated groups, and between two PGJ2-treated groups. The *p* values in red indicate the values that are significantly (< 0.05) different from DMSO-injected rats. *N* = 4 to 8 rats per group. **b** In the PGJ2-treated but not in the control (DMSO) rats, dopaminergic neuronal loss (TH+) in the ipsilateral SNpc (x-axis) inversely correlates with forelimb usage asymmetry (y-axis). X = number of injections (once per week)

Weekly PGJ2-microinfusions induced a gradual and significant bias toward use of the ipsilateral over the contralateral forelimb when compared to the DMSO controls (Fig. 3a). The Pearson correlation coefficient indicates a significant negative correlation between ipsilateral forelimb use and the number of ipsilateral TH+ cells in the SNpc in the PGJ2-treated rats (*r*^*2*^ = 0.6, *p* = 0.007; Fig. 3b, right panel). Such correlation was absent for the DMSO-treated rats (*r*^*2*^ = 0.047, *p* = 0.287; Fig. 3b, left panel).

These behavioral assessments together with the IHC data for the TH+ DA neurons (Fig. 2) strongly support that PGJ2 induces the development of PD-like pathology in a progressive manner over the period of 8 weeks post-treatment.

### PGJ2-treatment alters the morphological/functional properties of activated microglia in the rat SNpc, but does not change microglia total numbers in the same area

We analyzed the number of microglia in DMSO and PGJ2-treated rats. The intact contralateral SN served as the control for each rat. We thus evaluated and compared the activated microglia numbers for each rat, as a ratio between ipsilateral over contralateral sides.

**Fig. 4:**
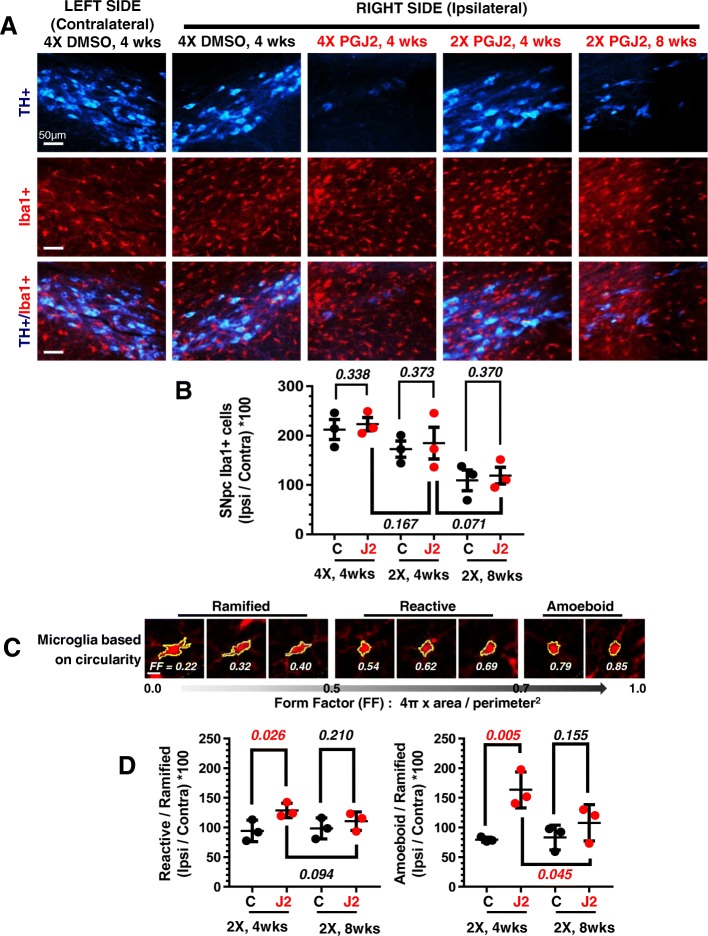
Successive PGJ2 microinfusions induce microglia morphological and functional changes detected post-mortem. **a** TH (blue, dopaminergic) and Iba1 (red, microglia) immunostaining shows a gradual loss of dopaminergic neurons and gain of activated microglia in the ipsilateral SNpc. Scale bar = 50 μm. **b** The overall numbers of microglia in the ipsilateral SNpc did not significantly differ between DMSO (control) and PGJ2–treated rats. **c** Microglia morphologic changes represented by the form factor (FF) calculated as 4π X area/perimeter^2^. **d** The number of reactive and amoeboid microglia significantly increased in the ipsilateral SNpc of rats receiving two (2X) PGJ2 injections at 4 weeks but not at 8 weeks post-injections, compared to controls (DMSO-treated). Values on the *y*-axis represent the ratios between the ipsilateral SNpc over the contralateral, normalized to the number of ramified microglia. Black circles, control, DMSO-treated rats; red circles, PGJ2-treated rats. Statistical significance was estimated with the Student’s *T* test to compare DMSO and PGJ2-treated groups, and between two PGJ2-treated groups. The *p* values in red indicate significant (*p* < 0.05) difference from DMSO-injected rats. *N* = 3 rats per group. X = number of injections (once per week)

Due to the surgical procedure, SN injections of DMSO alone increased microglia activity as assessed by Iba1 immunostaining. Compared to DMSO-treatment, PGJ2 did not significantly [*t*(4) < 0.449); *p* > 0.33] change the overall numbers of activated microglia, (Fig. 4a, b).

However, based on the FF values, we observed a significant rise in reactive [*t*(*4*) = 2.74; *p =* 0.026] and amoeboid [*t*(4) = 4.74; *p* = 0.005] microglia in PGJ2-treated rats compared to the DMSO-treated ones (Fig. 4d). The rise in reactive and amoeboid microglia was only detected in rats treated with two PGJ2 injections and sacrificed 4 weeks post-injection (Fig. 4d). The microglia rise was not apparent in the PGJ2-treated rats 8 weeks post-injection. The plotted values represent the ratio between ipsilateral over contralateral sides for each rat, normalized to the ramified microglia, which are the ones associated with normal physiological function.

### PGJ2-treatment increases astrocyte reactivity in the rat SNpc

Astrocytes in the brain become reactive under pathological conditions, including in neurodegenerative diseases [47]. Under these conditions, reactive astrocytes overexpress GFAP and undergo morphological changes (hypertrophy and process remodeling) [47].

**Fig. 5:**
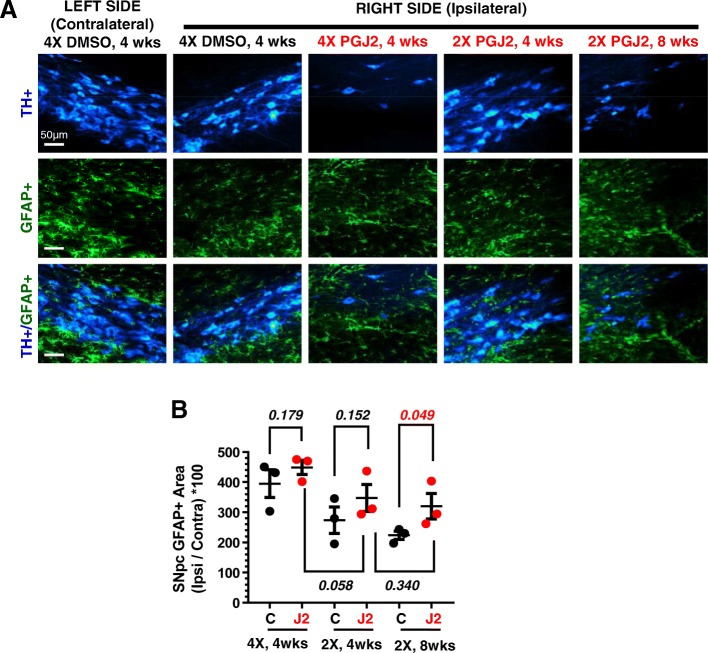
Successive PGJ2 microinfusions induce sustained astrocyte reactivity. **a** TH (blue, dopaminergic) and glial fibrillary acidic protein (GFAP, green, reactive astrocytes) immunostaining shows a gradual loss of dopaminergic neurons concurring with astrocyte reactivity in the ipsilateral SNpc. Scale bar = 50 μm. **b** PGJ2-treated rats show significantly increased astrocyte reactivity in the ipsilateral side of the SNpc compared to controls (DMSO-treated) at 8 weeks after two (2X) injections. Values on the *y*-axis represent the ratios between the ipsilateral SNpc over the contralateral. Black circles, control, DMSO-treated rats; red circles, PGJ2-treated rats. Statistical significance was estimated with the Student’s *T* test to compare DMSO and PGJ2-treated groups, and between two PGJ2-treated groups. The *p* value in red indicates significant (*p* < 0.05) difference from DMSO-injected rats. *N* = 3 rats per group. X = number of injections (once per week)

Our studies demonstrate that, compared to DMSO, rats that received two PGJ2 microinjections exhibited in the SNpc, a significant [*t*(4) = 2.14; *p* = 0.049] increase in astrocyte reactivity 8 weeks, post-injections (Fig. 5a, b). The astrocyte reactivity in the other groups of PGJ2-treated rats [2X, 4 weeks, *t*(4) = 1.18; *p* = 0.152; 4X, 4 weeks, *t*(4) = 1.04; *p* = 0.179] was non-significantly different from controls.

### Detection of intraneuronal aggregate-like inclusions of ubiquitinated proteins and phosphorylated (pS129) α-synuclein in SNpc DA neurons of rats treated with PGJ2

**Fig. 6:**
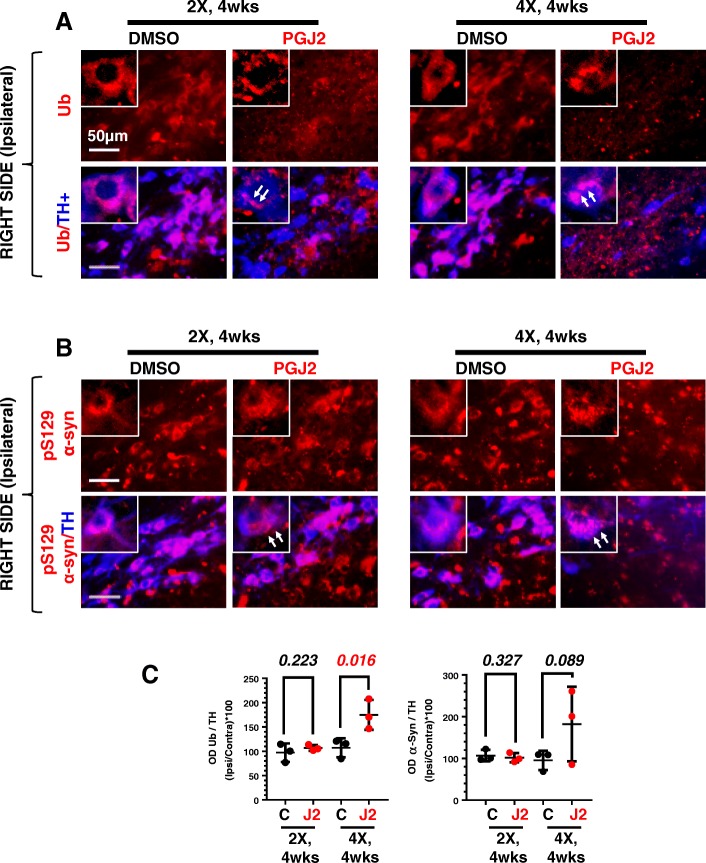
Successive PGJ2 microinfusions induce aggregate-like ubiquitinated protein (**a**) and pS129 α-synuclein (**b**) deposits in dopaminergic neurons. Immunostaining for ubiquitinated proteins (red in **a**), pS129 α-synuclein (red in **b**), and TH+ neurons (blue in **a** and **b**) at 4 weeks after two (2X) and four (4X) PGJ2 microinjections. Dopaminergic neurons in the SNpc of PGJ2-treated rats exhibit aggregate-like ubiquitin conjugates and pS129 α-synuclein neuronal inclusions (*white arrows*). Scale bar = 50 μm. **c** Values on the *y*-axis represent the optical density (OD) ratios between ipsilateral SNpc over the contralateral, normalized to the TH+ signal. Black circles, control, DMSO-treated rats; red circles, PGJ2-treated rats. Statistical significance was estimated with the Student’s *T* test to compare DMSO and PGJ2-treated groups. The *p* value in red indicates significant (*p* < 0.05) difference from DMSO-injected rats. *N* = 3 rats per group. X = number of injections (once per week)

The accumulation of proteinaceous intraneuronal inclusions known as Lewy bodies in SNpc DA neurons is one of the hallmarks of PD [48]. These inclusions contain ubiquitinated proteins [49] as well as α-synuclein phosphorylated at S129 (pS129 α-syn) [50]. We addressed this aspect of PD (Fig. 6a for ubiquitinated proteins, and 6b for pS129 α-syn, both quantified in 6c) by focusing on the TH+ DA neurons of the SNpc. Since our preceding studies already demonstrated the progressive nature of the PGJ2-induced rat model, for this analysis and all subsequent studies, we focused only on four experimental paradigms: rats treated with two injections or four injections of DMSO or PGJ2, and sacrificed 4 weeks post-injections.

Regardless of the number of PGJ2 injections, there was a shift from a diffuse distribution of ubiquitinated proteins and pS129 α-syn detected in TH+ neurons of DMSO-treated rats, to more of an aggregated appearance observed in the PGJ2-treated rats (white arrows, Fig. 6a, b). Moreover, we established that only treatment with four (not two) PGJ2 injections induced a significant [*t*(4) = 3.22; *p* = 0.016] elevation in the levels of ubiquitinated proteins in the few spared TH+ neurons (Fig. 6c, left panel). The levels of pS129 α-syn also increased under these conditions, almost reaching statistical significance [*t*(4) = 1.63; *p* = 0.089; Fig. 6c, right panel].

### Cell-type distribution and levels of key factors of the prostaglandin D2/J2 pathway

**Fig. 7:**
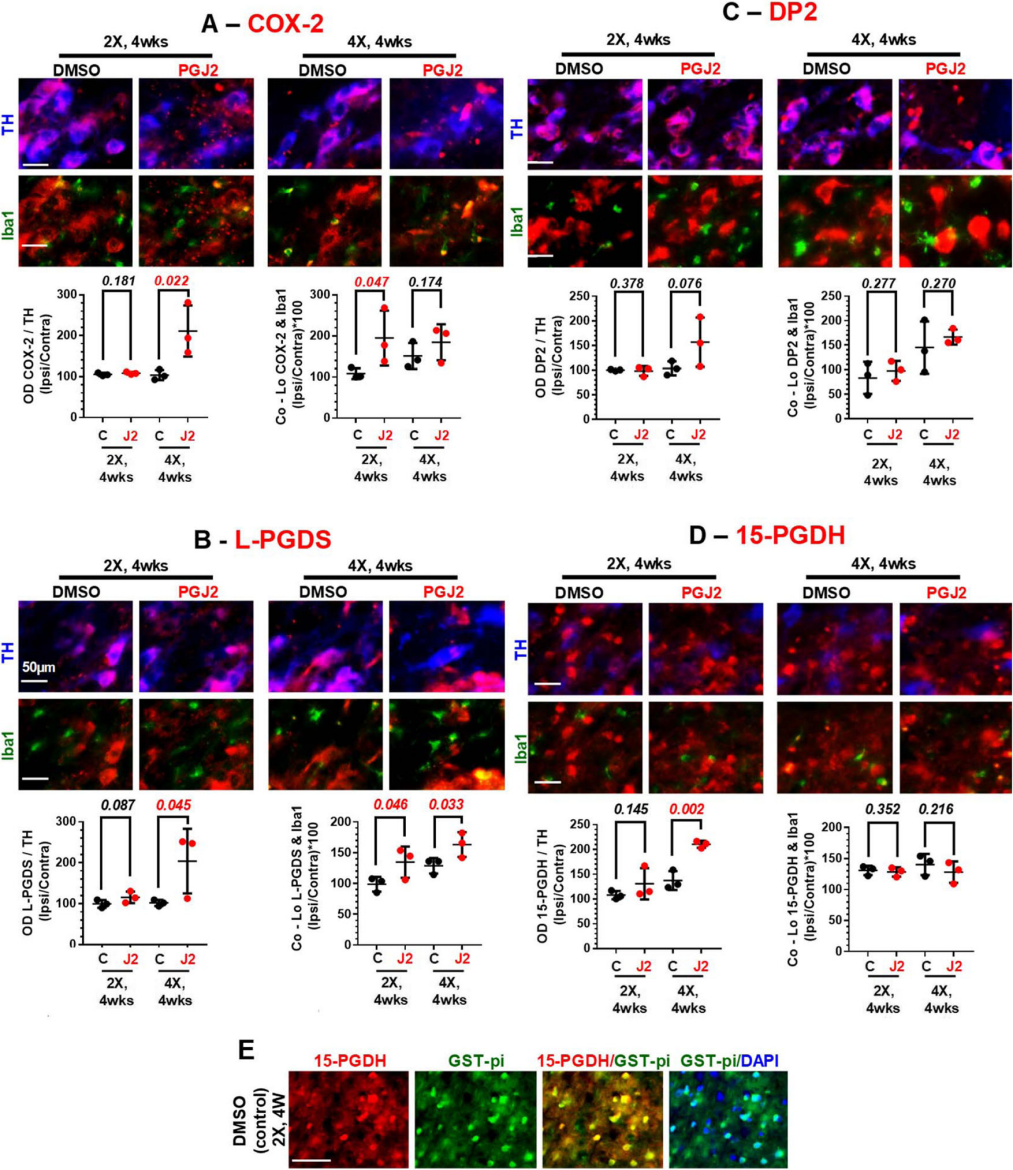
Effects of successive PGJ2 microinfusions on the following factors of the PGD2/J2 prostaglandin pathway: COX-2 (**a**), L-PGDS (**b**), DP2 receptor (**c**), and 15-PGDH (**d**), all in dopaminergic neurons and microglia, and 15-PGDH also in oligodendrocytes (**e**). Immunostaining for COX-2 (red in **a**), L-PGDS (red in **b**), DP2 (red in **c**), 15-PGDH (red in **d** and **e**), TH+ neurons (blue in **a**–**d**), Iba1+ microglia (green in **a**–**d**), and GST-pi+ oligodendrocytes (green in **e**) at 4 weeks after two (2X) and four (4X) PGJ2 microinjections. Scale bar = 50 μm. **a** COX-2 is significantly increased in dopaminergic neurons from rats receiving four (4X) PGJ2 injections compared to controls. Co-localization of COX-2 and Iba1 is greater in microglia from rats receiving two (2X) PGJ2 injections than in controls. **b** L-PGDS is significantly increased in dopaminergic neurons from rats receiving four (4X) PGJ2 injections than in controls. Co-localization of L-PGDS and Iba1 is greater in microglia from rats receiving two (2X) and four (4X) PGJ2 injections than in controls. **c** DP2 levels remain stable in dopaminergic neurons from all treatment groups, but DP2+ staining is almost absent in microglia. **d** 15-PGDH expression is increased in dopaminergic neurons from rats receiving four (4X) PGJ2 injections, but not in microglia. **e** 15-PGDH is highly expressed in SNpc oligodendrocytes from all groups of rats. Values on the *y*-axis represent the optical density (OD, normalized to TH, left graphs), or co-localization (normalized to Iba1, right graphs) ratios between ipsilateral SNpc over the contralateral. Black circles, control, DMSO-treated rats; red circles, PGJ2-treated rats. Statistical significance was estimated with Student’s *T* test to compare DMSO and PGJ2-treated groups. The *p* value in red indicates significant (*p* < 0.05) difference from DMSO-injected rats. *N* = 3 rats per group. X = number of injections (once per week)

Up to now, our findings corroborated that PGJ2-treated rats develop PD-like pathology in a progressive temporal manner. We wanted to take our in vivo investigation one step forward and address the impact of PGJ2 on the cell-type distribution and levels of key factors of the prostaglandin D2/J2 pathway observed during the 4-week window, including COX-2, the PGD synthase L-PGDS, the PGD2/J2 receptor DP2, and the prostaglandin dehydrogenase 15-PGDH (Fig. 7). We would like to highlight that the cell-type distribution and levels of these four factors have never been investigated under control or stress conditions in the SNpc in relation to PD, at least to our knowledge. Like for the ubiquitinated proteins and α-syn studies, we focused only on four experimental groups: rats treated with two injections or four injections of DMSO or PGJ2, and sacrificed 4 weeks post-injections.

We examined by immunocytochemistry, the distribution and levels of these factors in DA neurons and microglia in the SNpc by assessing the optical density ratio or co-localization of each of these factors with TH+ or Iba1+, respectively. There was no significant difference in the levels of these four factors in DA neurons (Fig. 7a–d, left) in rats that received 2× PGJ2 injections (red circles) compared to controls (DMSO-treated, black circles). In contrast, COX-2, L-PGDS, and 15-PGDH levels increased significantly in the DA neurons of PGJ2-treated rats that received four injections, when compared to controls (Fig. 7a, b, d, left). The DP2 receptor levels were stable in control and PGJ2-treated (4×) rats (Fig. 7c).

In regard to microglia, there was more variation in their responses to PGJ2-treatment. Starting with COX-2, we observed a rise in its levels in rats treated twice with PGJ2, but not in those treated four times with PGJ2, compared to controls (Fig. 7a, right). For L-PGDS, significant increases were detected in both groups of PGJ2-treated rats compared to controls (Fig. 7b, right).

Notably, DP2 receptors in microglia were almost non-existent or expressed at very low levels (Fig. 7c, right, and Table 1). When considering ipsilateral and contralateral SNpc separately, values representing the number of microglia exhibiting co-localization of the other three factors (COX-2, L-PGDS, 15-PGDH) with the microglia marker Iba1+, varied between 117 and 250 (not shown). However, for the DP2 receptor, these values ranged from 17 to 50 (Table 1); thus, they were 2 to 15 times lower. We suspect that in the microglia case, co-localization of DP2+/Iba1+ immunostaining is a paradox and is most likely due to the proximity of a microglia to a DA neuron.

**Table 1:** Low detection levels of DP2 and Iba1 co-localization in microglia. *N* = 3 rats per group. X = number of injections (once per week)

# of Co-Lo DP2 & Iba1 (SNpc)	2×, 4 weeks		4×, 4 weeks	
	Control	PGJ2	Control	PGJ2
Contralateral	29.7 ± 2.9	21.7 ± 2.3	17.3 ± 4.7	30.7 ± 2.7
Ipsilateral	24.3 ± 6.0	20.7 ± 0.3	22.3 ± 1.5	50.7 ± 3.9

The microglia levels of the prostaglandin dehydrogenase 15-PGDH did not change in any of the control or PGJ2-treated groups of rats (Fig. 7d, right). We found that this enzyme was expressed in another cell type, i.e., oligodendrocytes. The latter were detected in the SNpc of all groups of rats with the marker for mature oligodendrocytes, the pi form of glutathione-*S*-transferase (GST-pi, Fig. 7e).

### Ibuprofen prevents most of the PD-like pathology developed in PGJ2-treated rats

Ibuprofen (IBP) is an NSAID that inhibits COX-1 and COX-2, thus reducing the synthesis of all prostaglandins [1, 51]. We previously demonstrated that PGJ2 induces COX-2 upregulation in neuronal cells [25], potentially establishing a positive feedback loop between PGJ2 and COX-2. This positive feedback effect of PGJ2 on COX-2 could promote the transition from acute to chronic neuroinflammation and consequently promote progressive PD pathology.

**Fig. 8:**
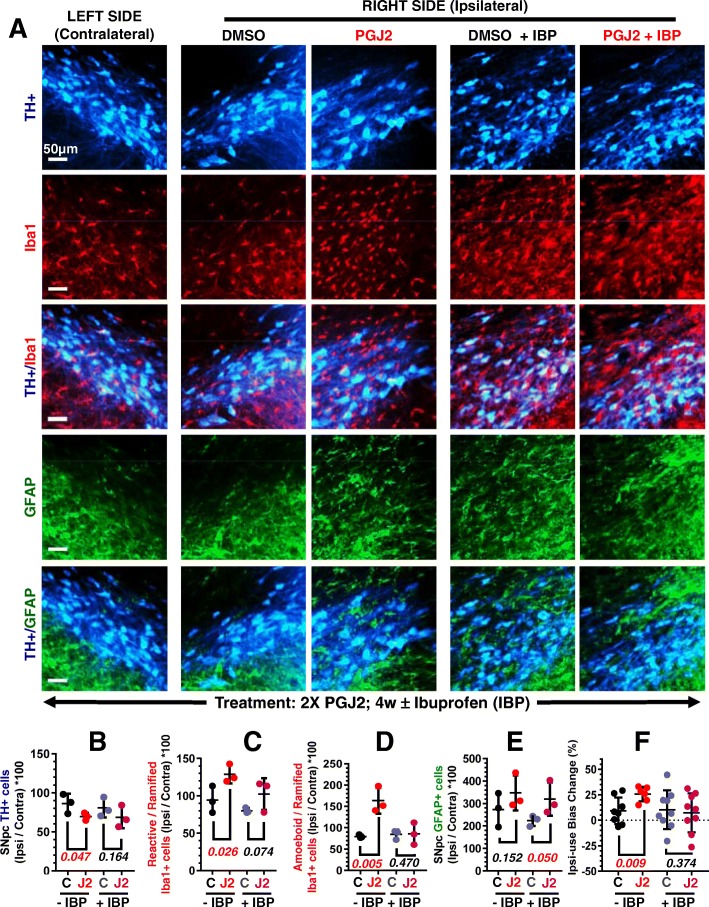
Ibuprofen (IBP) ameliorates the PD-like pathology induced by PGJ2 except for astrocyte activation. **a** Ibuprofen treatment starting the day after the first surgery reduces TH+ (blue, dopaminergic) neuronal loss and Iba1+ (red, microglia) activation, but not GFAP+ (green, astrocyte) reactivity in the SNpc at 4 weeks (4w) post two (2X) PGJ2-injections. Scale bar = 500 μm. **b** There was no statistical difference in TH+ DA neurons between ibuprofen-fed PGJ2-treated rats and ibuprofen-fed controls (DMSO-treated). The extent of PGJ2 damage was assessed by calculating the total number of TH + neurons (mean ± SEM) in the SNpc using unbiased stereology as described in Materials and Methods. **c**, **d** There was no statistical difference between the number of reactive (**c**) or amoeboid (**d**) microglia in ibuprofen-fed PGJ2-treated rats and ibuprofen-fed controls (DMSO-treated). **e** Astrocyte reactivity is significantly higher in ibuprofen-fed PGJ2-treated rats than in ibuprofen-fed controls (DMSO-treated). For A through E, *N* = 3 rats per group. Values on the *y*-axis represent the ratios between the ipsilateral SNpc over the contralateral (in **c** and **d** normalized to the number of ramified microglia). **f** There was no statistical difference in motor asymmetry between ibuprofen-fed PGJ2-treated rats and ibuprofen-fed controls (DMSO-treated). For **f**, *N* = 6 to 9 rats per group. Black and gray circles, control, DMSO-treated rats; red and pink circles, PGJ2-treated rats. Statistical significance was estimated with Student’s *T* test to compare DMSO and PGJ2-treated groups. The *p* values in red indicate significant (*p* < 0.05) difference from DMSO-injected rats

To determine the contribution of the cyclooxygenase pathway to the PGJ2-induced PD-like pathology in vivo, we administered IBP to the PGJ2-treated rats. The four groups of rats (2X DMSO ± IBP and 2X PGJ2 ± IBP) were analyzed at 4 weeks post the last PGJ2 treatment, with the cylinder test and immunochemistry. Preventive IBP-treatment starting the day after the first surgery ameliorated all PD-like pathology induced by PGJ2, except for reactive astrocytes (Fig. 8).

It is clear that IBP reduced DA neuronal loss (Fig. 8a, b), as well as reactive and amoeboid microglia rise (Fig. 8a, c, d) in the SN, and prevented the bias toward use of the ipsilateral over the contralateral forelimb (Fig. 8f). There was no statistical difference between DMSO and PGJ2-treated rats that were fed IBP in relation to these four parameters: numbers of TH+ cells (Fig. 8b), reactive (Fig. 8c) and amoeboid (Fig. 8d) microglia, and bias toward use of the ipsilateral over the contralateral forelimb (Fig. 8f). These results support the preventive effects of IBP in terms of PD-like pathology induced by PGJ2, a mediator of inflammation.

In contrast, IBP administration significantly [*t*(4) = 2.14; *p* = 0.05] increased astrocyte reactivity upon PGJ2-treatment, compared to DMSO-treatment (Fig. 8a, e). Thus, we show that PGJ2-treatment causes sustained astrogliosis, which is not alleviated by the IBP treatment (Figs. 5 and 8a, e). We should note that the role of astrocytes in PD pathology is ambiguous as activated astrocytes can produce both pro-inflammatory and anti-inflammatory factors, as well as neurotoxic and neurotrophic factors [52]. The types of reactive astrocytes that are induced by PGJ2 ± IBP remain to be investigated.

## Discussion

The frequent failure of potential new treatments in clinical trials suggests that current PD models may not exhibit the critical and/or complete set of PD traits experienced in humans. Other types of animal models relevant to PD need to be developed. Compelling evidence supports that neuroinflammation [1–5], and in particular the cyclooxygenase pathway, plays a central role in PD [6–8]. However, very few studies address how prostaglandin products of cyclooxygenases redirect cellular events to promote neurodegeneration in PD. In the current studies, we developed a non-transgenic rat model of neuroinflammation by microinfusing PGJ2 unilaterally into the right SNpc. The PGJ2-treated rats displayed progressive DA neuronal loss that correlates with motor deficits, as well as gliosis including increases in activated microglia and reactive astrocytes. The DA neurons in the SNpc developed aggregate-like ubiquitinated protein and pS129 phosphorylated α-synuclein deposits. All of these traits are characteristic of human PD-pathology.

**Fig. 9:**
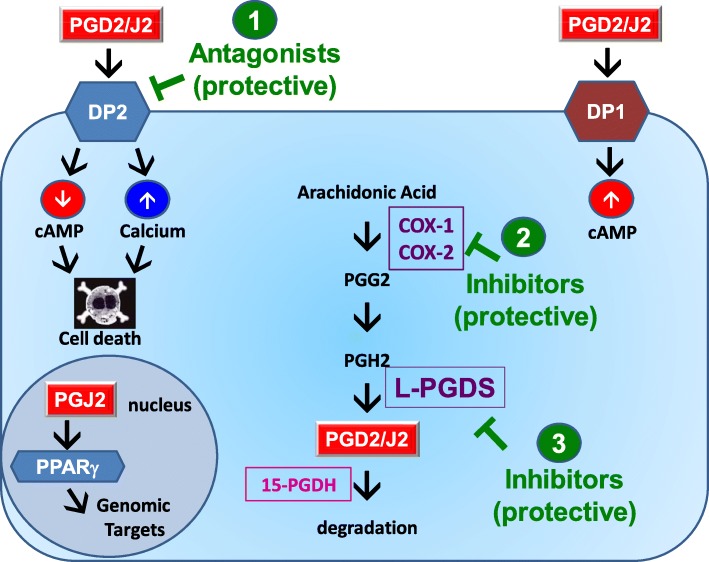
Scheme depicting novel and previously tested therapeutic agents (in green) that could have potential to treat PD. (1) DP2 receptor antagonists: in the SNpc, DP2 receptors were detected in dopaminergic neurons but not in microglia. DP2 receptor activation leads to a decrease in cAMP and an increase in calcium. These effects mediate neurotoxicity induced by DP2 receptor activation. Thus, DP2 receptor antagonists could potentially prevent DA neurodegeneration. (2) COX-inhibitors: Ibuprofen, a COX inhibitor, prevented many of the sequelae induced by the PGJ2 treatment. Our data support the epidemiological studies that report the beneficial effect of NSAIDs in lowering the risk of developing PD. (3) L-PGDS inhibitors: in the brain, this enzyme synthesizes PGD2 from PGH2. PGD2 and its metabolite PGJ2 can be neurotoxic. Thus, decreasing L-PGDS activity will lower PGD2 levels and could prevent or diminish the neurotoxic effects of PGD2/J2 on dopaminergic neurons. COX 1 and 2, cyclooxygenase 1 and 2; L-PGDS, lipocalin prostaglandin D synthase; 15-PGDH, 15-hydroxyprostaglandin dehydrogenase; PPARγ, peroxisome proliferator-activated receptor gamma; PET, positron emission tomography (see text for details)

This new PGJ2-induced rat model offers several advantages over other PD models. In the first place, we identified elements of the prostaglandin D2/J2 pathway that may be novel therapeutic targets for PD, downstream of the cyclooxygenase pathway (Fig. 9). Our studies show for the first time the distribution and levels of these elements in DA neurons and microglia of the SNpc. Although our studies do not address whether DP2 receptors or L-PGDS directly mediate the PGJ2 effects in the rat model, we propose that neuronal DP2 receptor antagonists and L-PGDS inhibitors could be novel drugs to prevent/diminish PD pathology associated with neuroinflammation (Fig. 9).

DP2 receptors bind PGD2 and PGJ2 with similar affinities (nanomolar range) [53, 54]. DP2 receptors are G*i*-protein-coupled-receptors, and their activation downregulates cAMP and increases intracellular calcium levels [55, 56]. We found that DP2 receptors are abundantly expressed in DA neurons of the SNpc, while almost lacking in microglia. Thus, PGD2/J2 released from activated microglia could lead to aggravated DA neuronal damage via DP2 receptor activation. On the other hand, DP2 inhibition could ameliorate neuronal dysfunction. In support of neuroprotection accomplished by blocking DP2 receptors, DP2 antagonists [CAY10471 and its BAY-u3405 (ramatroban) analog] were found to attenuate pain in a peripheral nerve injury rat model [57]. In addition, CAY10471 was shown to increase survival of rat hippocampal neurons exposed to aluminum [58]. In contrast, DP2 activation was shown to potentiate injury in rat hippocampal neuronal cultures and organotypic slices [7, 16]. Based on all of these findings, we propose that DP2 antagonists may protect DA neurons from the neurotoxic effects of activated microglia in PD, in particular SNpc DA neurons, which exhibit low calcium buffering capacity [59].

In regard to L-PGDS, this enzyme is responsible for most PGD2 synthesis in the brain and is one of the most abundant cerebrospinal fluid (CSF) proteins [60], representing ~ 3% of total CSF proteins [61]. We established that PGJ2-treated rats display increased L-PGDS levels, suggesting that L-PGDS inhibitors may protect neurons from PGD2/J2-induced PD-pathology. The following studies support this premise. Cortical neurons derived from L-PGDS null mice exhibited higher cell viability and less accumulation of ubiquitinated proteins in response to hypoxic conditions, than those derived from L-PGDS heterozygous mice [17]. L-PGDS was also identified as the major apoptotic factor in plasma of AD patients [62]. In addition, in vivo and in vitro studies demonstrated that L-PGDS promotes reactive gliosis related to brain pathology [63, 64]. Alterations in L-PGDS levels in the CSF were detected in at least 20 idiopathic PD patients compared to 100 controls [65]. These L-PGDS changes could indicate pathology at the cellular level with an impact on PGD2/J2 levels and also correlate with disease symptoms [65]. Based on all of these studies, we propose that L-PGDS could be considered a PD biomarker with predictive value [65] and could also be explored as a potential drug target for therapeutic intervention in neuroinflammation-driven PD.

Prostaglandins are unstable thus act as autocrine or paracrine ligands by exerting their effects near their sites of synthesis [66]. The enzyme 15-PGDH inactivates prostaglandins in the cytoplasm. Therefore, cyclooxygenases/synthases and 15-PGDH control prostaglandin cellular levels by opposite means. Although 15-PGDH metabolizes neuroprotective as well as neurotoxic prostaglandins, higher 15-PGDH levels may be beneficial under conditions where the effects of neurotoxic prostaglandins outweigh those of the neuroprotective ones [67]. We found that in PGJ2-treated rats, the levels of 15-PGDH expression in DA neurons seemed equivalent to those in control rats. In addition, we detected 15-PGDH in SNpc oligodendrocytes identified with GST-pi, the pi form of glutathione-*S*-transferase. The presence of 15-PGDH in oligodendrocytes may represent a protective mechanism to counteract the increased oligodendrocyte vulnerability to excitotoxic death associated with COX-2 up-regulation following, for example, glutamate receptor activation [68].

The second advantage offered by the PGJ2-induced rat model is related to animal models that recapitulate the disease phenotype with some degree of fidelity. These animal models should be valuable for developing and testing new or repurposed therapeutic agents as well as MRI and PET imaging tracers [69]. Our current studies show that when administered prior to symptom development, ibuprofen, which inhibits COX-1 and COX-2, prevented most of the PGJ2-induced PD-like pathology. This finding is important because it demonstrates that PGJ2-induced neurodegeneration is mediated in part by activation of the cyclooxygenase pathway that could lead to chronic inflammation. In addition, our data support that NSAIDs can be instrumental in preventing DA neurodegeneration associated with inflammation. The conflicting epidemiological and clinical results on the potential use of NSAIDs to treat PD could be due to variations in *(a)* length of treatment (longer in epidemiological and shorter in clinical studies), and/or *(b)* disease stage at the start of treatment (pre-pathology in epidemiological and post-pathology in clinical studies), reviewed in [70]. The PGJ2-induced rat model was also used to test a variety of PET tracers with the major intention of assessing in vivo the progression and spreading of neuroinflammation, (*manuscript in preparation*). These latter studies could help establish the optimal timeline for therapeutic intervention, to prevent PD pathology linked to neuroinflammation.

Thirdly, we show that the PGJ2-induced PD-like pathology in rats developed in a progressive manner, as it worsened with time up to 8 weeks post the second PGJ2 injection, the latest time-point that we analyzed. In animal models, the progressive nature of PD neurodegeneration is a hard-to-mimic aspect of this disorder. Toxin and pharmacologically induced PD models such as 6-hydroxydopamine, 1-methyl-1,2,3,6-tetrahydropiridine (MPTP), paraquat and rotenone exhibit motor symptoms, but they are often related to massive and rapid degeneration of the nigrostriatal pathway [71]. A similar phenomenon is observed in lipopolysaccharide (LPS)-mediated models of PD, used to address the link between neuroinflammation and PD pathology. LPS administered intracranially into the SN, striatum or globus pallidus causes DA neuronal loss within 24 h that persists at the same level up to 30 days, therefore not mimicking the progressive nature of PD pathology [72, 73]. Single systemic administration of LPS causes progressive dopaminergic neuronal loss, although microglia activation is not specific to the SN, being detected in a variety of brain regions [74].

Fourthly, we established that PGJ2 has similar neurotoxic effects in rats and mice, as we previously described for the PGJ2-induced mouse model that displayed comparable PD-like pathology [10, 11]. This is not the case for all drug-based PD models, some of which are species-specific. For example, rats are resistant to MPTP and the MPTP-sensitivity differs widely among various strains of mice [75]. Moreover, not all PD animal models exhibit DA neuronal degeneration and correlated motor deficits, as we detect in the PGJ2-induced rat model. For example, PD models based on genetic risk factors such as Parkin, PINK1 and LRRK2 show little or no nigrostriatal degeneration and motor symptoms [76]. Most PD models, transgenic and non-transgenic, exhibit some level of neuroinflammation usually detectable early and sometimes even pre-symptom development [77, 78]. It is thus advantageous to address novel targets of neuroinflammation, such as we do in the current study.

## Conclusions

Collectively, this is the first study to address the nigral dopaminergic and microglial distribution and levels of factors of the PGD2/J2 pathway in rodents. We focused on the PGJ2-induced rat model of neuroinflammation that exhibits progressive PD-like pathology, which is a hard-to-mimic aspect of this disorder. Comparisons between control and PGJ2-treated rats and the benefits of the ibuprofen treatment support that upregulation of COX-2 and L-PGDS may be important in the PGJ2-evoked PD-like pathology. Moreover, future mechanistic and pharmacological studies including this PGJ2-induced pre-clinical rat model will be highly valuable for identifying biomarkers and optimizing therapeutics, such as DP2 antagonists and L-PGDS inhibitors, which target neuroinflammation-driven PD circumventing the adverse side effects of cyclooxygenase inhibitors.

## Abbreviations

15d-PGJ2: 15-Deoxy-Δ^12,14^-PGJ2
15-PGDH: 15-Hydroxyprostaglandin dehydrogenase
AD: Alzheimer’s dementia
AP: Anterior-posterior
COX: Cyclooxygenase
CSF: Cerebrospinal fluid
DA: Dopaminergic
DMSO: Dimethyl sulfoxide
DP2: Prostaglandin D2/J2 Receptor 2
DV: Dorsal-ventral
FF: Form factor
GABA: Gamma-aminobutyric acid
GFAP: Glial fibrillary acidic protein
GST-Pi: Pi form of glutathione-*S*-transferase
i.p.: Intraperitoneal
Iba1: Ionized calcium-binding adapter molecule 1
IBP: Ibuprofen
IHC: Immunohistochemistry
L-PGDS: Lipocalin-PGDS
LPS: Lipopolysaccharide
LRRK2: Leucine-rich repeat kinase 2
ML: Medial-lateral
MPTP: 1-Methyl-1,2,3,6-tetrahydropiridine
NeuN: Neuronal nuclei
NSAID: Non-steroidal anti-inflammatory drug
O.D.: Optical density
PD: Parkinson’s disease
PET: Positron emission tomography
PG: Prostaglandin
PGD2: Prostaglandin D2
PGH2: Prostaglandin H2
PGJ2: Prostaglandin J2
PINK1: PTEN (phosphatase and tensin homolog)-induced putative kinase 1
PPARγ: Peroxisome proliferator-activated receptor gamma
pS129: Phosphorylated at residue S129
SN: Substantia nigra
SNpc: Substantia nigra pars compacta
SNpr: Substantia nigra pars reticulate
TBI: Traumatic brain injury
TH: Tyrosine hydroxylase
